# Dental Colleges and Honorary Degrees

**Published:** 1859-03

**Authors:** 


### DENTAL COLLEGES AND HONORARY DEGREES.

We take it for granted, that the profession generally feel a great interest in the success of Dental Collegiate education. It is certainly through those institutions that dental science must receive its greatest development for good. We have now three institutions of this kind, which appear to be somewhat stable, and their success for the last few years, has been such as to impress on the profession, much of its present character.

So long as there are old and meritorious practitioners, who have entered the profession before their organization, it should be expected that occasionally such should receive the highest honors of the profession. This we regard as right and proper; and if the power thus granted them is judiciously exercised, none should complain. But we are reminded, that two other institutions of a like character, have been started, and from various circumstances, such as their location, etc., they necessarily had to discontinue their organization. We have been advised, that one of these, although "dead yet speaketh," and that its voice has again and again been heard in the form of an honorary degree, from *one* of its *former* Faculty.

Now, we regard as very doubtful, the propriety of this course. We can not, indeed, realize how this can be done—such institutions have, or should have, a Board of Trustees, and, that one man can well be the *Board of Trustees*, its President, and all the Faculty, is rather more than we can fathom.

We should like to see a diploma signed—John Jmith, D. D. S., Prof. of Operative Dentistry; John Smith, D. D. S., Prof. of Mechanical Dentistry; John Smith, D. D. S., Prof. of Anatomy; John Smith, D. D. S., Prof. of Chemistry; John Smith, D. D. S., Prof. of Pathology; John Smith, President of the Board of Trustees; and John Smith; Secretary of—conferring the title of D. D. S., on Elijah Smith, student.

This is said to be a fast age, and this, verily, appears to be the fastest *stage* of the age.

We hope, for the credit of the profession, and those engaged in it, that this is not so. Do you know any thing of the matter? Yours, truly, ONE OF THE PROFESSION.

[We have heard an *inkling* of it.—ED.]

